# Experimental dataset to assess the structural performance of cracked reinforced concrete using Digital Image Correlation techniques with fixed and moving cameras

**DOI:** 10.1016/j.dib.2023.109703

**Published:** 2023-10-20

**Authors:** Andreas Sjölander, Valeria Belloni, Viktor Peterson, Jonatan Ledin

**Affiliations:** a Division of Concrete Structures, Department of Civil and Architectural Engineering, KTH Royal Institute of Technology, Stockholm, Sweden; b Geodesy and Geomatics Division, Department of Civil, Constructional and Environmental Engineering, Sapienza University of Rome, Rome, Italy; c Statik och Form AB, Stockholm, Sweden

**Keywords:** Dataset for DIC and DIC-enhanced measurements of concrete cracks, Modelling of existing cracks in concrete, Dataset for material models of reinforced concrete, FE modelling of existing cracks, Structural assessment of cracked concrete

## Abstract

The infrastructure is in many countries aging and continuous maintenance is required to ensure the safety of the structures. For concrete structures, cracks are a part of the structure's life cycle. However, assessing the structural impact of cracks in reinforced concrete is a complex task. The purpose of this paper is to present a dataset that can be used to verify and compare the results of the measured crack propagation in concrete with the well-known Digital Image Correlation (DIC) technique and with Crack Monitoring from Motion (CMfM), a novel photogrammetric algorithm that enables high accurate measurements with a non-fixed camera. Moreover, the data can be used to investigate how existing cracks in reinforced concrete could be implemented in a numerical model.

Therefore, the first potential area to use this dataset is within image processing techniques with a focus on DIC. Until recently, DIC suffered from one major disadvantage; the camera must be fixed during the entire period of data collection. Naturally, this decreases the flexibility and potential of using DIC outside the laboratory. In a recently published paper (Belloni et al., 2023), an innovative photogrammetric algorithm (CMfM) that enables the use of a moving camera, i.e. a camera that is not fixed during data acquisition, was presented. The imagery of this dataset (Sjölander et al., 2023) was used to verify the potential of this algorithm and could be used to validate other approaches for non-fixed cameras. The second potential area is structural engineering. The data can be used to verify non-linear material models used in finite element (FE) software to simulate the structural response of reinforced concrete. In particular, the data can be used to investigate how existing cracks should be modelled in a FE model.

The dataset presented in this paper includes data collected from a three-point bending test performed in a laboratory environment on uncracked and pre-cracked reinforced concrete beams. Structural testing was performed using a displacement-controlled set-up, which continuously recorded the force and the vertical displacement of a centric placed loading piston. First, the response of three uncracked beams was recorded. Thereafter, photos of the resulting cracks were taken, and a detailed mapping was presented. Material properties for the concrete, e.g., compressive strength, are presented together with testing of the tensile capacity of the reinforcement and a compressive test of the soft fiber boards used at the support to ensure good contact between steel and concrete. Then, the structural response of the pre-cracked beams was tested. During this test, four fixed cameras were used to monitor the crack propagation at different locations on the beam. Images are presented at the start of the load sequences and at pre-defined load stops during the testing. Hence, the crack opening captured in the images can be correlated to the force-displacement data. Moreover, a non-fixed camera was used to capture additional imagery at the location of each fixed camera.

Specifications Table

**Table:** 

Subject	Civil and Structural Engineering
Specific subject area	Three-point bending tests of pre-cracked reinforced concrete beams monitored with digital cameras
Type of data	Images of cracks Monitoring of crack propagation using imagery Structural test data
How the data were acquired	The data was acquired in a laboratory using a displacement-controlled test with a 250 kN MTS machine. During testing, the piston's required force and corresponding displacement were measured at 25 Hz. Four fixed cameras were used to monitor the development of cracks: three GoPro Hero 9 with a resolution of 20 megapixels, and one Canon EOS 5D Mark II with a CMOS sensor with a resolution of 21 megapixels. An iPhone SE 2 with a 12 MP sensor was used as a non-fixed camera. Images are presented at specific load-stops. The correlation between time and image file for each camera is reported in an Excel spreadsheet.
Data format	.dwg .pdf .jpg .png .txt .xlsx
Description of data collection	Data were collected in a laboratory environment. Four fixed cameras were continuously monitoring the displacements. A data logger with 25 Hz frequency was used to record the force and displacement of a centric placed steel piston. To ensure synchronization of data, imagery of cracks was presented at pre-defined load levels.
Data source location	KTH Royal Institute of Technology, Stockholm, Sweden
Data accessibility	Repository name: Mendeley Data Data identification number: 10.17632/z3yc9z84tk.3 Direct URL to data: https://data.mendeley.com/datasets/z3yc9z84tk/3
Related research article	*V. Belloni, A. Sjölander, R. Ravanelli, M. Crespi, A. Nascetti.* Crack Monitoring from Motion (CMfM): Crack detection and measurementusing cameras with non-fixed positions*. Automation in Construction, 156. 2023.* *doi:*https://doi.org/10.1016/j.autcon.2023.105072

## 1 Value of the Data

• From a computer vision and image processing perspective, this data can be used to compare the results of the measured crack propagation using a camera with a fixed position to the results obtained from a camera with a non-fixed position over time. Therefore, this data can be used to validate and compare new approaches for processing images acquired from different positions over time.
• The use of GoPro cameras in the test can provide an interesting case study for evaluating the potential of these sensors for measurements in the laboratory environment. Finally, considering the distance used in the test between the cameras and the beam to monitor, this dataset can be adopted to design and perform more challenging test configurations for data acquisition.
• From a structural engineering perspective, this data can be used to verify numerical models of reinforced concrete. More specifically, the data can be used to verify modelling techniques for existing cracks. The dataset also includes images to compare crack evaluation between a numerical model and experimental tests.

## 2 Objective

The data was collected to support an original research paper which presents a novel photogrammetric algorithm to measure the propagation of cracks using a camera with non-fixed position over time. For this purpose, crack propagation was measured at four locations using fixed cameras. Testing was paused at pre-defined load levels in which additional images were captured at the four locations with a camera with no fixed position. A comparison between calculated crack widths with the fixed and moving cameras is presented in the research paper by comparing the performance of the well-established DIC technique [1] and the innovative CMfM approach [2].

Additional data was captured during testing to extend the possible use of this dataset to include structural assessment and verification of FE models. The data collected can be used to verify non-linear material models to simulate the behaviour of reinforced concrete. Moreover, the precise documentation of the location and geometry of existing cracks before testing, and the structural response of the damaged beam, makes it possible to investigate how existing cracks in the concrete should be modelled in FE software.

## 3 Data Description

**Fig. 1:**
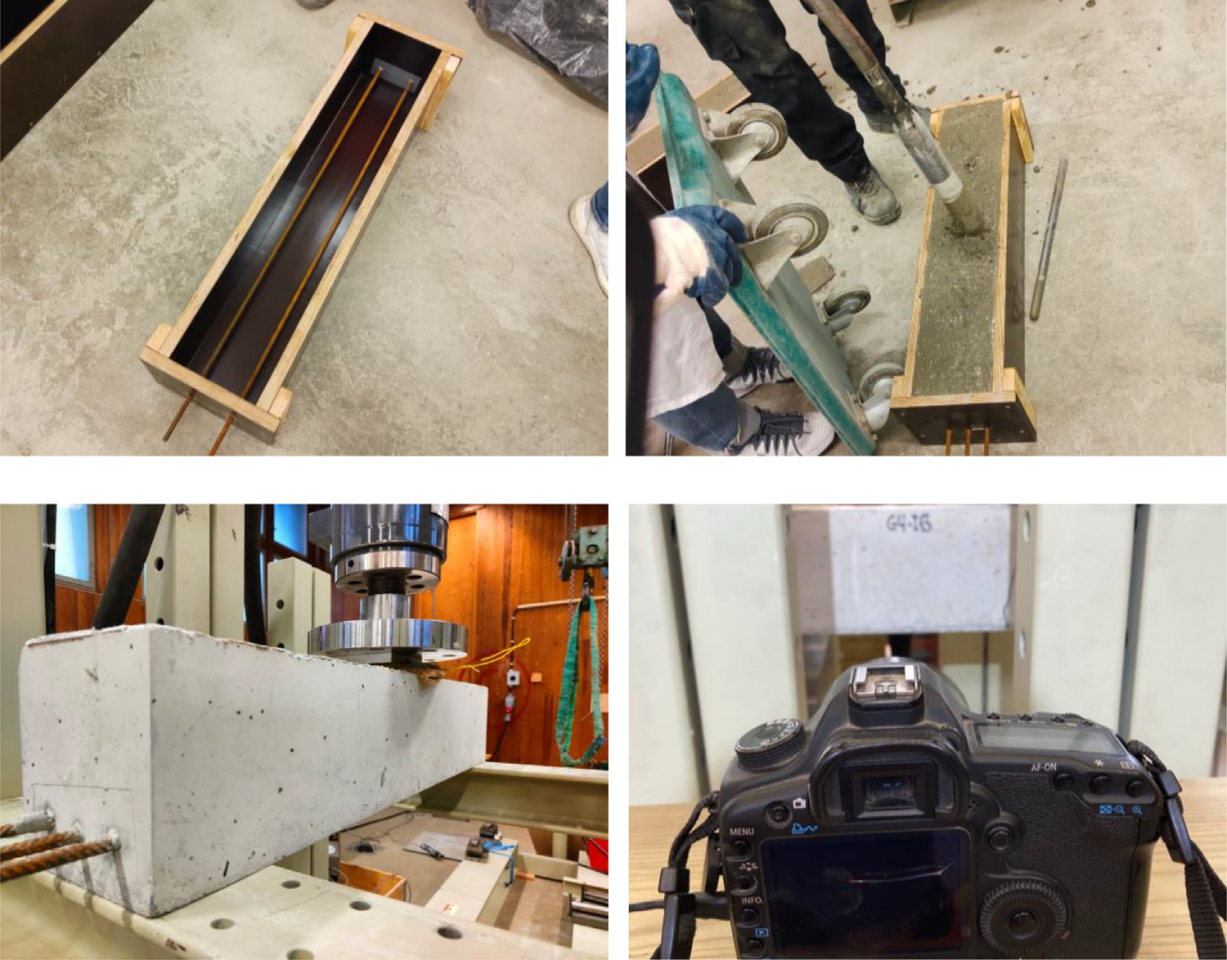
Overview of casting (top) and testing (bottom).
• Test Data: contains a plot showing the force-displacement for the two tests, i.e. uncracked and cracked concrete as well as the filtered data from all tests in .txt format.
• Overview: includes images from the casting and testing.
• Beam 4: Data from the test. See the description below.
• Beam 5: Data from the test. See the description below.
• Beam 6: Data from the test. See the description below.
  ○ Existing Cracks - DWG: 2D drawings of the beam crack patterns prior to testing in .dwg format. Drawings for cracks at each side as well as the top and bottom of the beam.
  ○ Existing Cracks - Images: original images of the existing cracks prior to testing in .jpg format. Images on the top, bottom and both sides. One reference image with a metric ruler is included for each side. An example of existing cracks as .dwg and .jpg is shown in Fig. 2.

The published dataset is structured in seven main folders:

• Material: contains data for structural testing of reinforcement, concrete and fiber board placed under the piston to ensure good contact between steel and concrete during testing.
• Geometry: presents the beam's geometry and the reinforcement arrangement. In Fig. 1, an overview of the test set-up is shown.

**Fig. 2:**
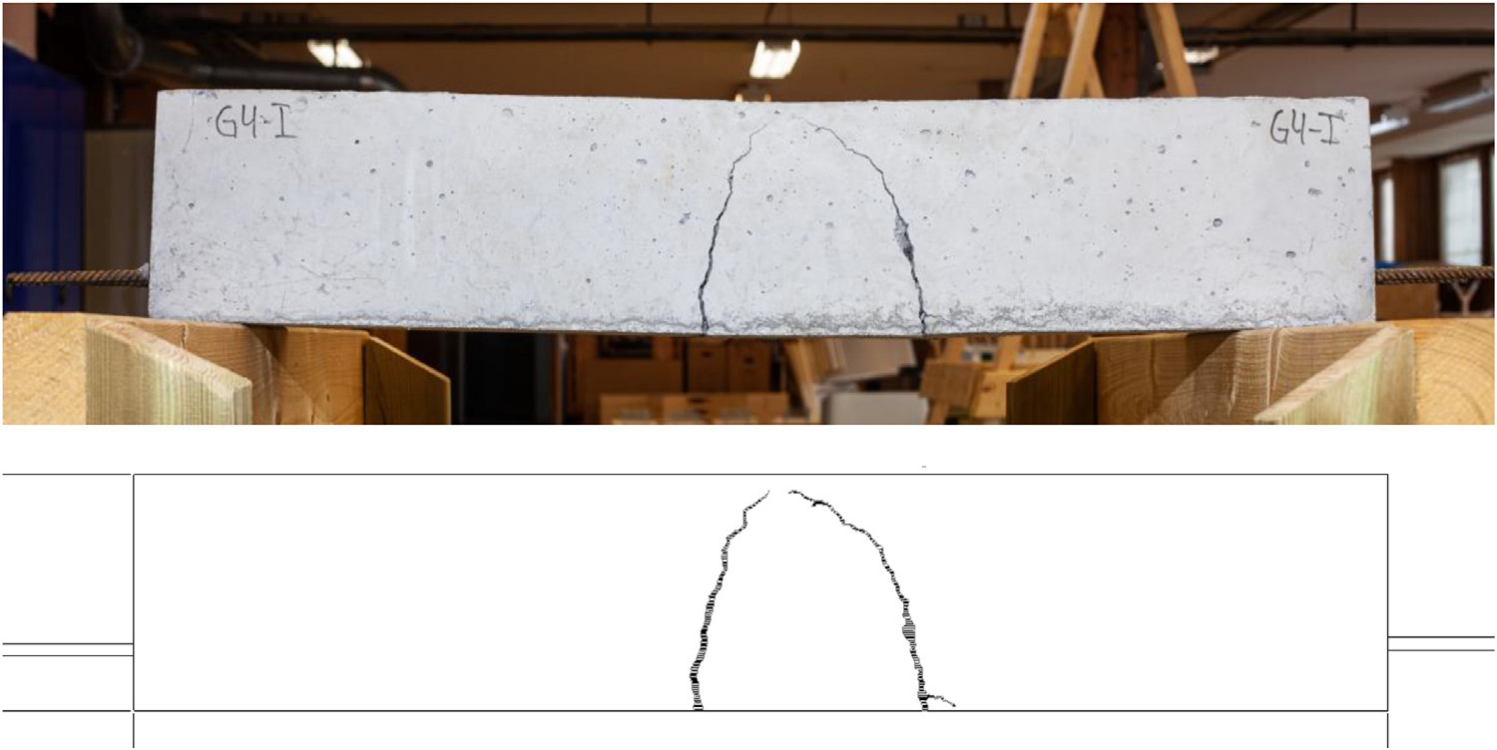
Existing cracks on Beam 4 side I shown as .jpg (top) and .pdf (bottom).
  ○ Existing Cracks - PDF: printed drawings from CAD-drawings of the crack patterns.
  ○ Test Data: contains an Excel Spreadsheet with practical information and the raw data files from the MTS machine. This includes how data was acquired, i.e. the distance between each camera and the beam, as well as the time when each camera started. No synchronization exists between the data logger measuring force-displacement and the cameras. Therefore, a clock was first started. Then, the start time for each camera and the test system was noted in the spreadsheet to synchronize the data in the post-processing.
  ○ Gx-IA-Fixed: images captured with a fixed GoPro camera on the area labelled as IA. The letter x refers to the beam number, i.e. 4,5,6.
  ○ Gx-IB-Fixed: images captured with a fixed Canon camera on the area labelled as IB.
  ○ Gx-IIC-Fixed: images captured with a fixed GoPro camera on the area labelled as IIC.
  ○ Gx-IID-Fixed: images captured with a fixed GoPro camera on the area labelled as IID.
  ○ Gx-XY-Free: images captured with a non-fixed iPhone camera. Images were captured from all the XY positions, i.e. IA, IB, IIC and IID.
    Each folder includes one or two reference images which contain a ruler to provide a metric reference for the pixel-to-millimetre conversion of the pixel size. In Fig. 3, examples of images captured with the GoPro and Canon cameras are shown.

**Fig. 3:**
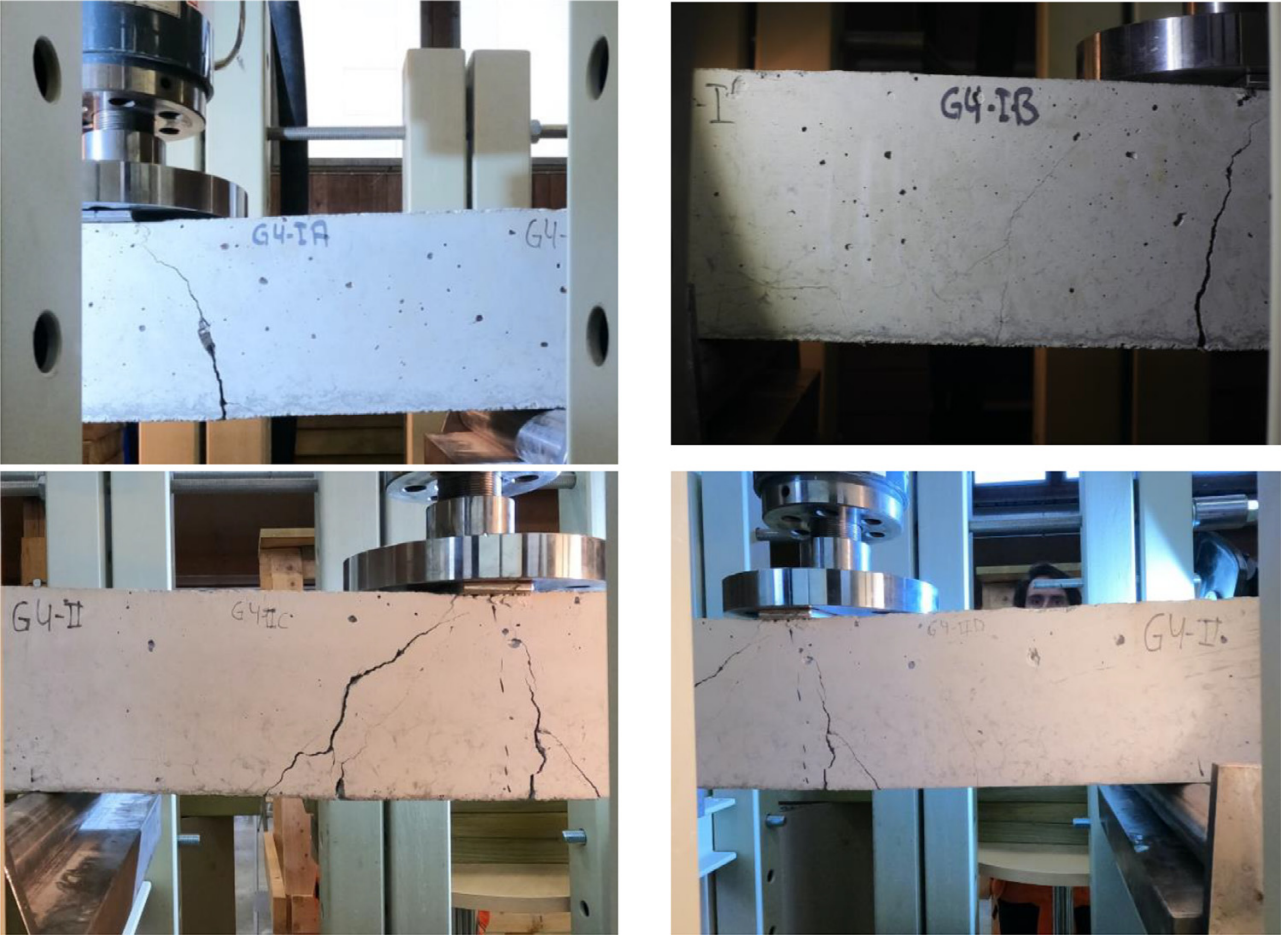
Images from the experiment showing the cracking on the four different locations of Beam 4 captured at *t* = 588 s.
  • Filtered Data: contains a plot showing the force-displacement and the time-displacement for the two tests, i.e. uncracked and cracked concrete as well as filtered data from the test in .txt format. In the filtered data, the time signal has been adjusted to match the time indicated for each photo. This was done based on the time noted in the Excel Spreadsheet in the Test Data folder. The data contains the following columns:
    ○ Num: number of datapoints.
    ○ Time (s): time in seconds counted from the start of test. See the Excel sheet for reference regarding the start of each camera.
    ○ PosAbs (mm): absolute position of the piston during the test.
    ○ Pos(mm): relative displacement of the piston during test, i.e. piston was reset to zero upon contact with the beam. Thus, it describes the centric displacement of the beam.
    ○ Pos (%): -.
    ○ Load (kN): corresponding load measured in kN.
    In Fig. 4, the force-displacement curves for the uncracked and pre-cracked concrete beams are shown.
    • Final Cracks: contains images of the cracks after the final test.

**Fig. 4:**
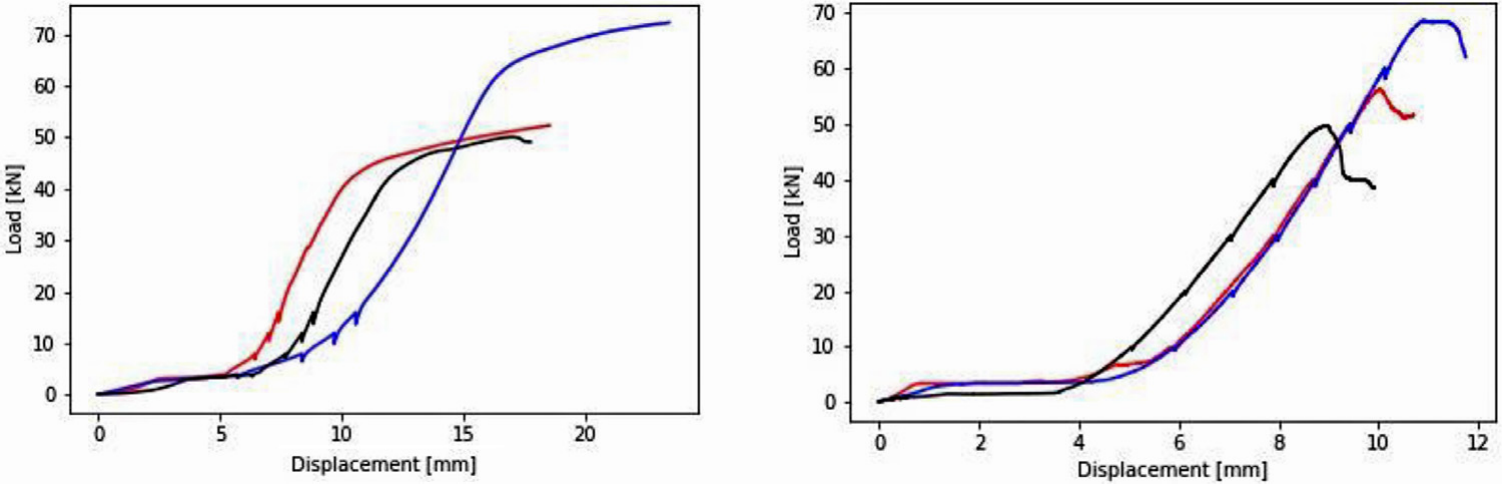
Force-displacement curves for uncracked beams (left) and pre-cracked beams (right).

## 4 Experimental Design, Materials and Methods

**Casting of Concrete:** Three concrete beams were cast in a laboratory environment using the concrete mix provided in the Material folder. Two different reinforcement arrangements were used, two bars or three bars. The geometry of the beams is provided in the Geometry folder.

**Curing of Concrete:** After casting, the beams were covered with plastic sheets until the next day, i.e. approximately 24 hours. Thereafter, the beams were cured in water for 27 days. The beams were removed from the water the day before testing, i.e. approximately 24 hours before testing, and kept indoors in the laboratory until testing.

**Material Testing:** Beam 5-6 were cast from one batch of concrete, while Beam 4 was from a separate mix. One 100 × 100 × 100 mm cube was cast for each beam, i.e. Cube 4–6, to test the compressive strength. The same recipe was used for all beams. The reinforcement was tested using a uniaxial tensile test. Slipping occurred between the anchors and the reinforcement bar before the displacement started. Hence, the exact strain in the reinforcement is difficult to determine. The fiber boards were tested using an MTS machine. The dimensions of the boards were 150 × 50 × 14 mm, i.e. the same size used during testing. A total of three boards were tested. For boards 2 and 3, the unloading curve of the plate is also presented.

**Fig. 5:**
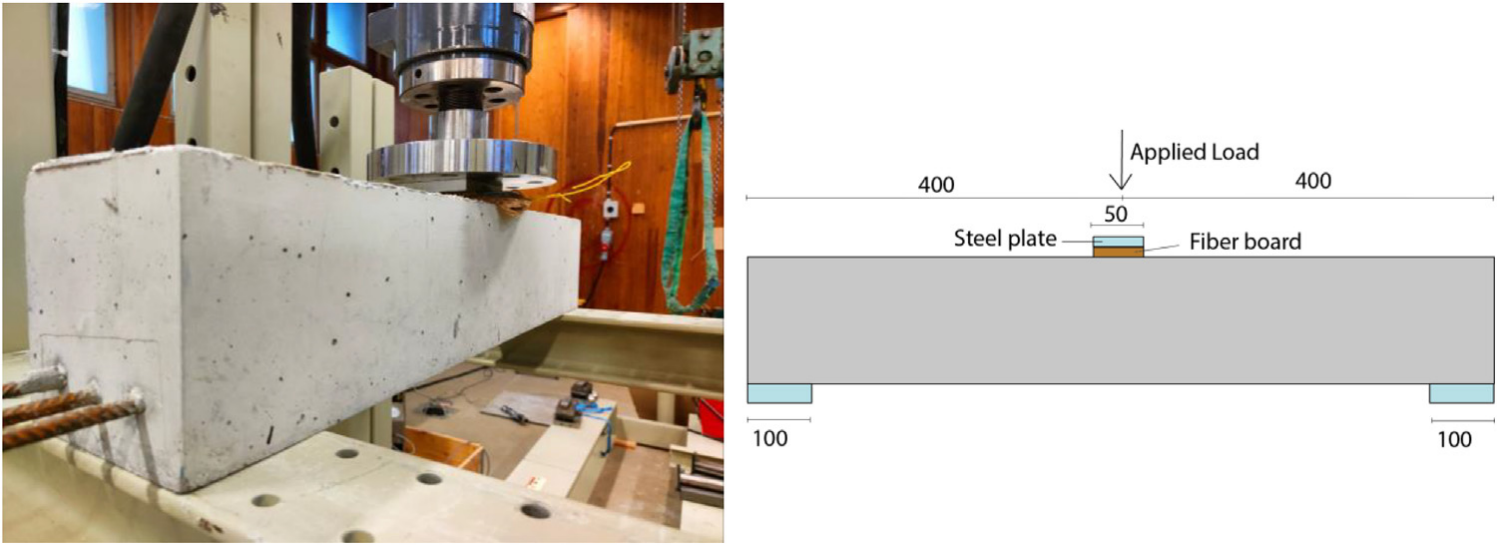
Test set-up for testing of uncracked concrete beams.

**Fig. 6:**
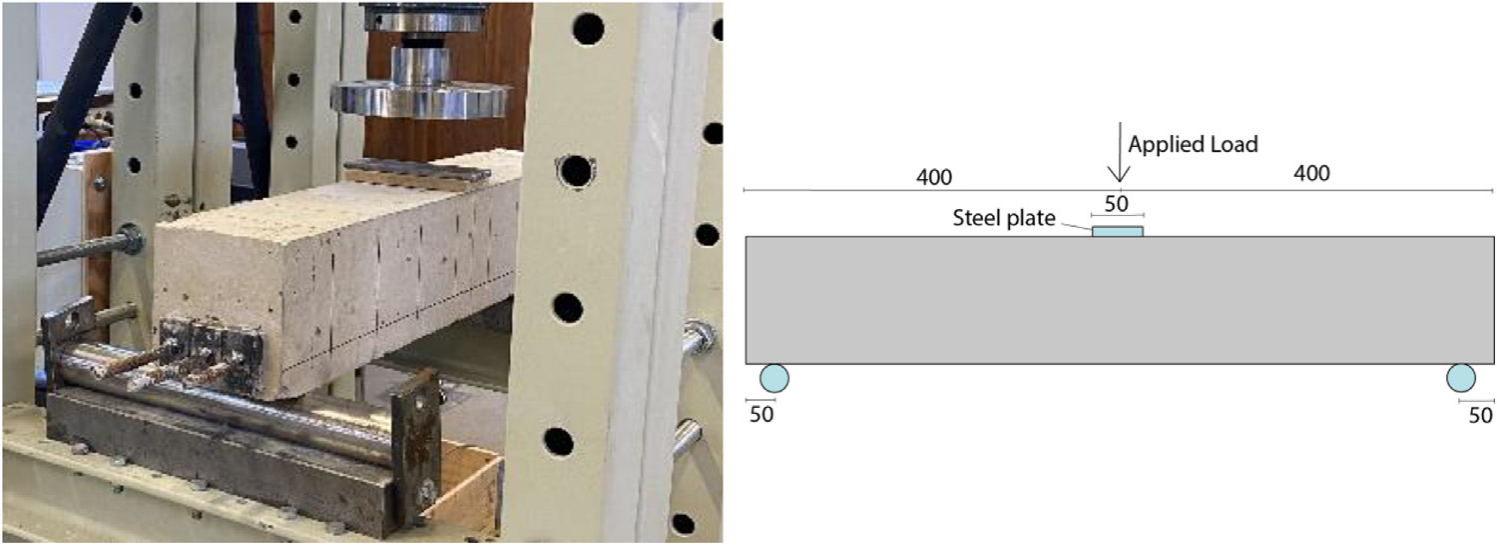
Test set-up for testing of pre-cracked concrete beams.

**Experimental set-up:** Three-point bending was used and the set-up for the experiment is shown in Figs. 5 and 6 for the uncracked beams and the pre-cracked beam, respectively. The uncracked beams were supported by a 100 mm wide steel beam. The beams were not rigidly connected to the test plate. Hence, small rotations of the support were allowed. Loading was applied through a 10 mm thick steel plate placed on top of a 14 mm thick fiber board to ensure good contact and more uniformly distributed load along the width of the beam. The rate of displacement was 2 mm/min throughout the test. The pre-cracked beams were placed on circular steel supports placed on top of the same steel beams as used for the uncracked beams. The load was applied in the same way as for the test of uncracked concrete beams.

**Fig. 7:**
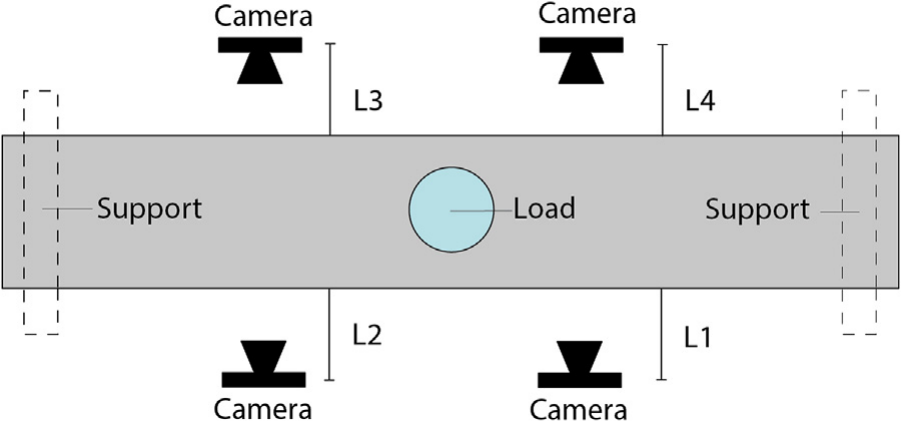
Top view of the experimental set-up and location of cameras together with measured distance for each camera.

**Data collection set-up:** Testing was performed using a displacement-controlled MTS machine with a capacity of 250 kN MTS. During testing, the piston's required force and corresponding displacement were measured with 25 Hz. Four cameras were placed to monitor different sections of the beam, see Fig. 7. As a metric reference for the physical pixel size, a reference image is included for each location. The distance between the centre of the camera and the surface of the beam was measured for each test and noted in the spreadsheet in the Test Data folder. The centric placed piston is visible in all collected images and can be used to calculate the location of the monitored area. The fixed cameras were fixed during the entire test-series. One image from the fixed camera is presented from all the positions for each load-stop. For the moving cameras, three images were taken of the same area, but from slightly different positions, during each load-stop. The position of the moving camera was random.

Images with the Canon camera were acquired using a lens with a fixed focal length of 50 mm. Images with the GoPro Hero9 camera were acquired using “Linear Field of View” which should reduce the effect from the fish eye lens and be equivalent to a focal length between 19 and 39 mm.

## Limitations

None.

## Ethics Statement

The proposed data does not involve any human subjects, animal experiments, or data collected from social media platforms.

## CRediT authorship contribution statement

**Andreas Sjölander:** Conceptualization, Methodology, Writing – original draft, Resources. **Valeria Belloni:** Conceptualization, Methodology, Writing – review & editing. **Viktor Peterson:** Writing – review & editing, Resources. **Jonatan Ledin:** Writing – review & editing, Resources.

## Data Availability

Monitoring of structural performance of cracked reinforced concrete using DIC and CMfM (Original data) (Mendeley Data).

## Appendix Supplementary materials

Image, application 1

## References

[1] Belloni V., Ravanelli R., Nascetti A., Di Rita M., Mattei D., Crespi M. (2019). py2DIC: A New Free and Open Source Software for Displacement and Strain Measurements in the Field of Experimental Mechanics. Sensors.

[2] Belloni V., Sjölander A., Ravanelli R., Crespi M., Nascetti A. (2023). Crack Monitoring from Motion (CMfM): crack detection and measurement using cameras with non-fixed positions. Autom. Construct..

